# Blood Flow Disturbance and Morphological Alterations Following the Right Atrial Ligation in the Chick Embryo

**DOI:** 10.3389/fphys.2022.849603

**Published:** 2022-04-14

**Authors:** Maha Alser, Huseyin Enes Salman, Azza Naïja, Thomas Daniel Seers, Talha Khan, Huseyin Cagatay Yalcin

**Affiliations:** ^1^Biomedical Research Center, Qatar University, Doha, Qatar; ^2^Department of Mechanical Engineering, TOBB University of Economics and Technology, Ankara, Turkey; ^3^Petroleum Engineering Program, Texas A&M University, Doha, Qatar

**Keywords:** congenital heart defects, ventricle, chick embryo, right atrial ligation, ultrasound, micro-CT, hemodynamics, computational fluid dynamics

## Abstract

Collectively known as congenital heart defects (CHDs), cardiac abnormalities at birth are the most common forms of neonatal defects. Being principally responsible for the heart‘s pumping power, ventricles are particularly affected by developmental abnormalities, such as flow disturbances or genomic defects. Hypoplastic Right Heart Syndrome (HRHS) is a rare disease where the right ventricle is underdeveloped. In this study, we introduce a surgical procedure performed on chick embryo, termed right atrial ligation (RAL) for disturbing hemodynamics within the right heart aiming in order to generate an animal model of HRHS. RAL is a new surgical manipulation, similar to the well-studied left atrial ligation (LAL) surgery but it induces the hemodynamic change into the right side of the heart. After inducing RAL, We utilized techniques such as Doppler ultrasound, x-ray micro-CT, histology, and computational fluid dynamics (CFD) analysis, for a comprehensive functional and structural analysis of a developing heart. Our results displayed that RAL does not induce severe flow disturbance and ventricular abnormalities consistent with clinical findings. This study allows us to better understand the hemodynamics-driven CHD development and sensitivities of ventricles under disturbed flows.

## Introduction

The cardiac muscle is a complex organ that forms and begins to function early in the embryonic stages. During the early stages, the heart starts beating while synchronously remodeling (Chien et al., 2009; Lindsey et al., 2014). Heart development relies on multiple factors, including hemodynamics, which is the dynamics of blood flow within the heart and blood vessels (Yalcin et al., 2010a). Over many years, researchers highlighted the pattern of the hemodynamic flow in the embryonic circulatory system and supported its importance in cardiac looping, primitive chamber formation and alignment, cardiac septation and maturation as well as the evolution of vessels (Boon et al., 2007; Groenendijk et al., 2007; Yalcin et al., 2011; Bharadwaj et al., 2012; Yalcin, 2014). It is well known that disturbed hemodynamics can lead to the progression of congenital heart defects (CHDs) (Chivukula et al., 2015). Studies have shown that mechanical stress applied on the myocardium affects ventricular development and remodeling, endothelial cell organization, and signaling (Fisher et al., 2000; Hove et al., 2003; Van der Heiden et al., 2006). However, the mechanisms behind these changes remain poorly understood.

Ventricular defects are amongst the most severe CHDs. Left and right ventricles develop in a similar manner during development and their sizes are similar during these initial stages. Before septation, a newly developing four-chamber heart is in a serial configuration where the primitive LV is directly connected to inflow and the primitive RV is directly connected to outflow. The pre-septation stage is known to be the developmental time period where most CHDs initiate. Hypoplastic left heart syndrome (HLHS) is a very severe disease responsible for 25 to 40% of neonatal deaths (Hoffmann et al., 2017). A similar CHD type affecting the right ventricle is the hypoplastic right heart syndrome (HRHS), which is less severe than HLHS (Van Der Hauwaert and Michaelsson, 1971) but it is still considered lethal as the right side of the heart is responsible for circulating the blood from and to the lungs through the pulmonary circulation. Briefly, HRHS is a rare cyanotic CHD characterized by underdevelopment of tricuspid and/or pulmonary valves and the right ventricle with right to left shunting through an inter-atrial communication. Clinically, the abnormality varies in severity considerably according to the level of the hypoplasia and the extreme forms are associated with tricuspid and/or pulmonary atresia as suggested by Van Der Hauwaert and Michaelsson (1971).

At early stages, the easy optical access to avian tissues allows researchers to monitor the blood flow where a surgical alteration may develop similar disease phenotypes with the human fetus. Many studies highlighted the efficiency of chick embryos to investigate the influence of disturbed hemodynamics on heart development (Yalcin et al., 2010, 2011; Henning et al., 2011; Alser et al., 2021; Salman et al., 2021) since chicken share identical molecular and morphological cascades of heart development with human (Groenendijk et al., 2007; Katritsis et al., 2007). Left atrial ligation (LAL) is a well-established surgical technique performed upon chick embryos. LAL has been shown to significantly modify early cardiac development and generate a HLHS phenotype in the animal (Sedmera et al., 1999). Like for LAL microsurgery, right atrial ligation (RAL) indeed allows the increase of the blood flow to the left ventricle since the blood flow circulating in the right atrioventricular valve is reduced by the RAL. In this intervention, a surgical knot is tightened around the developing right atrium of the chick embryo to constrict the blood flow in the right side of the heart. This practice reduces the atrial volume capacity and decreases the effective volume of the right atrium.

By measuring blood flow velocity waveforms, Doppler ultrasound allows the assessment of cardiac output, peripheral resistance index and mean arterial pressure (Groenenberg et al., 1989). This advanced *in vivo* imaging method has been widely used to assess changes in embryonic hemodynamics under normal and stressed conditions during the early developmental stages (Midgett and Rugonyi, 2014; Yalcin et al., 2017; Benslimane et al., 2020; Salman and Yalcin, 2020). Another technique widely considered to access structural damages in different organ tissues is histology (Naïja et al., 2017, 2018; Arman and Üçüncü, 2020). In chick embryos, Sedmera et al. (2002) and Tobita and Keller (2000) highlighted the importance of the histopathological technique in detecting abnormalities of heart macroelements. In the same context, micro-CT is a more detailed technique allowing the analysis of heart macrostructures. It enables the generation of 3D heart volumes with high-resolution visualization of the perfused hearts (Doost et al., 2020).

As known, blood flow exerts shear stress on the endocardium, a frictional force per unit area that is tangential to the surface and that arises due to the blood motion (Courchaine et al., 2018). As demonstrated by Groenendijk et al. (2007), changes in wall shear stress (WSS) resulting from changes in blood flow velocities lead to alteration of cardiovascular development.

Bioengineering is being associated to biomedical studies in order to develop systems and devices helping to resolve clinical issues. Computational fluid dynamics (CFD) is a numerical method using physically governing flow equations to calculate parameters, not possible to be calculated directly (Salman and Yalcin, 2021). This technique is able to calculate 3D and 4D blood flow velocities and distributions of WSS on the endocardium using data extracted from micro-CT and ultrasound (Courchaine et al., 2018) and other imaging modalities (Midgett et al., 2015; Boselli and Vermot, 2016; Lee et al., 2016).

To better understand the mechanism by which RAL affects the cardiogenesis of chick embryos, we performed RAL microsurgery on embryonic day 4 (ED4) hearts. The hemodynamic analyses are performed after microsurgery (ED5 and ED7). We first followed the mean flow waveforms in the AV canal using Doppler ultrasound. Then, we analyzed and quantified morphological changes in RAL hearts before (ED5) and after septation (ED7) using histology and micro-CT methods respectively. To confirm the minor effect of RAL on heart development, we performed the CFD modeling to compute shear stresses on the endocardium. With this study, we tried to give an explanation to answer the question: WHY HRHS IS LESS SEVERE THAN HLHS.

## Materials and Methods

### Embryonic Chick Culture

Fertilized eggs (*Gallus gallus*) were cultured and maintained as described previously (Benslimane et al., 2019). In summary, the eggs were incubated at 37.5°C, 60% humidity, with a continuous rocking for 72 h, embryonic day 3 (ED3), HH21 in a stationary incubator (GQF 1500, digital professional). On ED3, eggs were opened using the following procedure: initially each egg was laid horizontally for few minutes. On the blunt end of the egg, a small hole was made using surgical scissors to allow albumin extraction. Then, a syringe needle was inserted vertically to extract 5 ml of albumin. On the top, the shell was taped, and a small window was made to open the egg. Subsequently, the opening was sealed using an adhesive seal. The opened eggs were kept in a portable incubator (GQF 1588 – Genesis Hova-Bator) set on 37.5°C and 60% humidity and stored prior to surgical intervention. The culture method is explained in details in our previous work (Benslimane et al., 2019).

### Right Atrial Ligation

**FIGURE 1:**
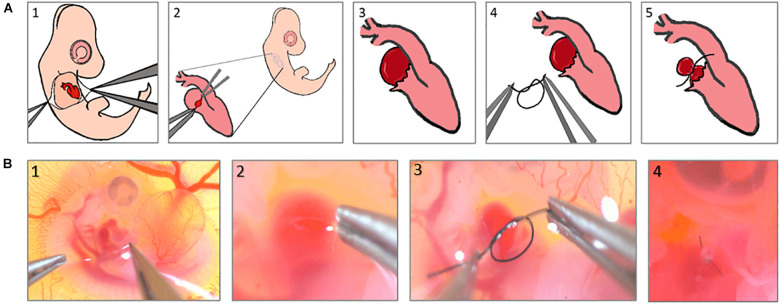
Right atrial ligation surgery steps, **(A)** schematics to show the steps of the surgery, **(B)** Real microscopic pictures of the surgery steps.

In this study, the procedure was conducted at ED4 corresponding to Hamburger-Hamilton 23 (HH23) stage, on a clean bench using UV-sterilized tools. Under a stemi-508 stereo microscope, the chorionic and allantoic membranes are opened with two pairs of sharp forceps (Figures 1A1,B1), allowing the right atrium to be located. The epicardial membrane around the right atrium is then opened (Figures 1A2,B2), and a pre-prepared 10-0 ethilon surgical suture is gently tightened around it, reducing the original primitive RV volume to ∼50% (Figures 1A3,A4,B3). Finally, the egg is re-sealed and returned to the incubator for further use.

### Processing Doppler Ultrasound Waveforms

Doppler ultrasound performed in the present work is well explained in our previous study (Benslimane et al., 2019). Briefly, embryos are cultured in-ovo by making a window enabling visualization of the heart and accessing with an ultrasound probe. This step is called “opening.” Embryos are then kept in the bench-top incubator with fixed temperature and humidity parameters (37°C and 72% respectively). Depending on the studied stage, embryos are placed in a dry block heater set to 37 °C for environmental stabilization. A drop of warmed Tyrode is added to make a bridge between the heart and the probe (20 MHz) which is placed onto a WPI 3301 micromanipulator. Signal analysis is performed using Indus mice Doppler system, adapted for chick embryos. For AV measurements, the animal is flipped so that the left side is on top. For OFT, the animal is on its normal orientation, residing on its left, the right side is on top. For maximum signal acquisition, the probe is oriented to be parallel with the blood flow. Once doppler waveforms are acquired, they are traced automatically with the software provided by the manufacturer, and the graph is converted to Microsoft Excel for further analysis.

In this work, the flow velocities in the atrioventricular (AV) canal are compared between RAL and control hearts using ultrasound-based Doppler echocardiography measurements. A total of 18 chick embryos (9 samples for RAL hearts and 9 samples for control hearts) were used consisting of nine specimens for each group to determine the mean flow waveforms in the AV canal at the ED5, HH27 (1 day after the surgical interference). For each embryo, a sample recording which covers one complete cardiac cycle is selected and the moment of peak flow rate is identified for each cardiac cycle. Then, the time length of each recording is normalized according to the peak flow moments, such that all the peak values of nine different recordings within each group overlap after the normalization. In the final step, the mean flow profiles are determined by averaging the nine normalized recordings for each group. Average flow profiles were used to define the velocity boundary condition of CFD simulations.

### Histological Analysis for Morphological Assessment

Histology was used as a tool to assess morphological changes induced by RAL. Controls and RALs were analyzed at two different time points: the pre-septation stage corresponding to ED5, HH27, and the septation stage corresponding to ED7, HH30. For each group and time point, three samples were analyzed. Hearts were extracted from the animals under a Zeiss Stemi-508 stereo microscope, fixed in 4% PFA (ChemCruz) for 24 h on a tube rotator, serially dehydrated and wax infiltrated. Then, the hearts were embedded in paraffin wax and sectioned into 5 μm thick sections using automated rotary microtome (Thermofisher HM35S, United States), with the sections placed on glass slides upon on a slide warmer (45°C) for 1 h. The slides were then stained with Hematoxylin and Eosin and observed under an optical microscope, Zeiss Stemi-508 stereomicroscope.

### Microfil Cast Creation

Microfil casts were created for the control and RAL embryonic hearts at 2 timepoints: ED 5 (HH27) and ED7 (HH30). We perfused Microfil (Flow-Tech, Carver, MA, United States) into the cardiac cavity and the surrounding vascular lumens using glass capillary micro-needles (Butcher et al., 2007). For each group, three microfil casts were created and analyzed.

The technique was detailed in our previous paper published by Salman et al. (2021). In summary, Microfil solution is prepared and injected into the pumping heart of the embryonic chick. This solution polymerizes into a cast, preserving the cardiac chambers as they are. The body is then fixed in PFA 4% and preserved in at 4 °C for x-ray micro CT imaging.

### Micro-CT Imaging

Embryos were loaded into x-ray transparent capillaries and imaged using a Thermo-Fisher Heliscan Mk1 x-ray microcomputed tomography scanner using a space-filling helical scan protocol and an energy range of 80–90 keV. Aluminum filters were employed to minimize beam hardening artifacts, with the reconstructed image resolution ranging between 7.6–14 μm (governed by the diameter of the sample chamber used). Finally, reconstructed voxel images were denoised using the edge-preserving non-local means filter of Buades et al. (2005). These 3D geometries were subsequently used to quantify ventricular chamber volume and AV valve orifice sizes of control and RAL embryos.

### Processing Micro-CT Images for Morphological Measurements

The datasets are then reassembled into 3D geometry using Mimics (Materialise, Leuven, Belgium) software package. The heart anatomy was segmented as previously described (Butcher et al., 2007). Morphological measurements are performed using the generated 3D models of ED5 and ED 7 control and RAL hearts. ANSYS Workbench DesignModeler module was used to calculate the heart chamber volumes. Embryonic hearts were digitally sectioned into pieces according to the dimensions of the chambers. In addition to the heart chamber volumes, CFD simulations were employed to investigate the hemodynamics in the left and right AV canals and also to calculate the volume averaged vorticities within the analyzed hearts. The vorticity is a measure that indicates the circulation and flow rotation within the flow domain.

### Computational Fluid Dynamics Model Generation and Simulations

Computational fluid dynamics represents a group of numerical methods used to investigate fluid transport phenomena across numerous scales of interest. Obtaining *in-vivo* flow measurements from a developing heart is challenging, due to its limited dimensions and highly dynamic nature. Image-based CFD modeling enable realistic flow conditions to be simulated using the micro-CT images of a developing heart, circumventing the above-mentioned challenges. In this study, the computational meshes amenable to perform CFD simulations were obtained from micro-CT voxel images of chick embryonic hearts using Materialize Mimics: a processing software package for 3D design and modeling. After obtaining the 3D model of the embryonic heart, boundary conditions were applied at the inlet and outlet of the model domain. The inlet boundary is defined using a time-dependent velocity profile. The outlet was set as a pressure boundary with zero-gauge pressure. The remaining boundaries in the flow domain were set as walls with a no slip condition. The assumption of no slip condition guarantees that the flow on the wall is always stagnant. The Doppler ultrasound measurements were employed to determine the inlet boundary conditions of the CFD simulations. By performing numerous trials, the proper inlet flow rates were determined for the models by adjusting the measured echocardiography flow profiles at AV canals. With the adjustment of inlet flow boundary conditions, both the CFD simulations and Doppler echocardiography measurements resulted in the same peak flow rates in the AV canals. This approach validates the numerical findings of the CFD simulations by comparing the results with *in-vivo* measurements. After setting the appropriate boundary conditions, the flow domain was discretized in the 3D space, which is known as meshing. At the final step, the governing flow equations were solved for each mesh element and the results were post-processed to visualize the determined flow streamlines and flow variables such as velocity, pressure, and wall shear stress (WSS). The governing flow equations are known as the Navier-Stokes and continuity equations, which are given in Eqs 1 and 2, respectively.


(1) $$\rho_{f}\frac{\partial \text{v}}{\partial t}+\rho_{f}\left(v\right)\cdot \nabla \text{v}-\nabla \cdot \tau_{f}=0$$

(2) ∇⋅v=0

In Eq. 1, the fluid velocity vector is defined by **v**; time is defined by *t*; fluid stress tensor is defined by τ_*f*_, and mass density of the blood is defined by ρ_*f*_. The continuity equation guarantees that the inlet mass flow is always equal to the outlet mass flow in the problem domain.

Computational fluid dynamics simulations were performed for three control and two RAL chick hearts at ED5, which is 1 day after the RAL interference. ED5 was selected as the time point for CFD studies as we expect induction of disturbed hemodynamics at this pre-septation stage. CT images of the selected embryonic hearts were used to generate 3D embryonic geometries using Materialize Mimics segmentation software package. Using this software, the surface quality of 3D models was improved by smoothing algorithms and the inner holes and sharp corners which may result in instability during the CFD simulation were eliminated from the mesh.

In the simulations, we modeled the left and right atrium, left and right ventricles, and left and right atrioventricular (AV) canals. We employed a static heart geometry during the cardiac cycle with a constant volume and fully opened valve configuration. The chamber contraction is modeled by removing approximately half of the heart chamber volumes. In comparison to the distant borders of the heart chambers, the areas closer to the AV canals experience less contraction and remain relatively static. Therefore, we removed the highly dynamic regions of the heart chambers by removing approximately half of the chamber volumes. As consequence, we predict that our models will give the most accurate results during the peak flow conditions, particularly around the AV canals.

**FIGURE 2:**
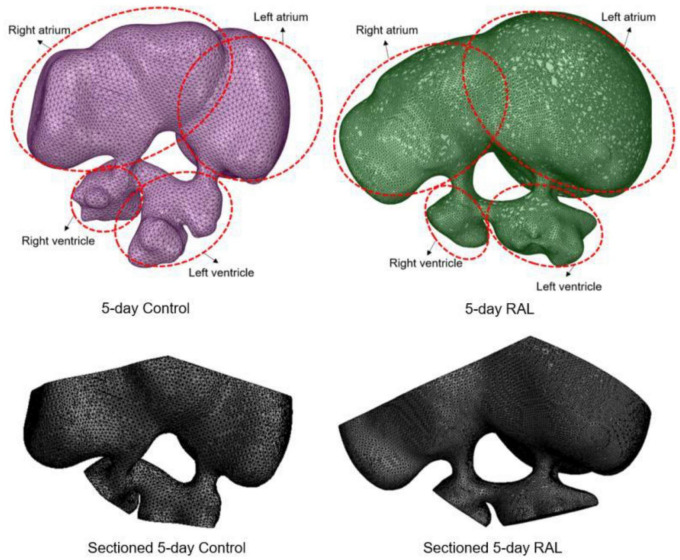
3D models of ED5 control and ED5 RAL hearts. Sectioned 3D models are shown at the bottom to account for the volumetric contraction in the heart chambers. About half of the volume of the chambers was removed in order to apply inlet and outlet boundary conditions in the CFD models.

Computational fluid dynamics meshes were sectioned and modified in order to define inlet and outlet boundary conditions. About half of the volume of the heart chambers was sectioned and removed to account for the volumetric reduction imposed by the contraction of the heart. In Figure 2, the modified 3D models of RAL and control hearts are presented after completing the sectioning operations described above. The inlet flow is applied as a function of time on the sectioned surface of the left and right atrium. The outlet boundaries are selected as the sectioned surfaces of the left and right ventricles. For obtaining mesh independent results with high accuracy, the dimensions of the mesh elements should be sufficiently small. The characteristic length of a tetrahedral element was used as approximately 5 × 10^–5^ m. When the total number of mesh elements was higher than 500,000, the relative difference in the peak AV canal velocity was found to be less than 2% between two independent meshes. Therefore, the hemodynamic results of a mesh composed of 500,000 tetrahedral elements were considered as sufficiently accurate. The generated meshes are composed of mixed tetrahedral elements with four displacement nodes at the corners of each tetrahedral prism and one pressure node in the center of each element. In order to improve the quality of the mesh, inflation layers were used on the wall boundaries for increasing the solution accuracy, enabling the development of a boundary layer at the heart wall.

In CFD simulations, blood was modeled with a constant mass density of 1,060 kg/m^3^. The blood viscosity was modeled as shear rate dependent. In this way, the viscosity of the blood changes with the flow conditions and the shear environment. This approach is known as non-Newtonian fluid characteristics, and it is a more realistic way for modeling the blood compared to using a constant viscosity model. We used the model of non-Newtonian power law based on previous studies on avian blood viscosity (Yalcin et al., 2011; Salman et al., 2021) by employing consistency index of 1.7 × 10^–5^, power index of 0.708, minimum viscosity limit of 1 centipoise, and maximum viscosity limit of 100 centipoise. The time length of a complete cardiac cycle was 0.5 s and one complete cardiac cycle was simulated using a total of 40-time steps with increments of 0.0125 s.

### Statistical Analysis

Statistical analysis was performed using GraphPad Prism 6 statistical software and Excel. Distributional was determined using the D’Agostino-Pearson normality test. Parametric data were analyzed using one way-ANOVA with Sidak post-hoc for multiple comparisons test while nonparametric data were analyzed using the Kruskal–Wallis test with Dunn’s post-hoc test. The correlation test was accomplished by Pearson correlation test for parametric data and Spearman correlation test for non-parametric data. In all analyses, a *p*-value of less than 0.05 was considered significant. Peak velocity, time-averaged velocity, ejection time, chamber sizes, valve sizes, WSS levels, and vorticity levels were compared among different groups.

## Results

### The Effect of Right Atrial Ligation on AV Canal Velocities

**FIGURE 3:**
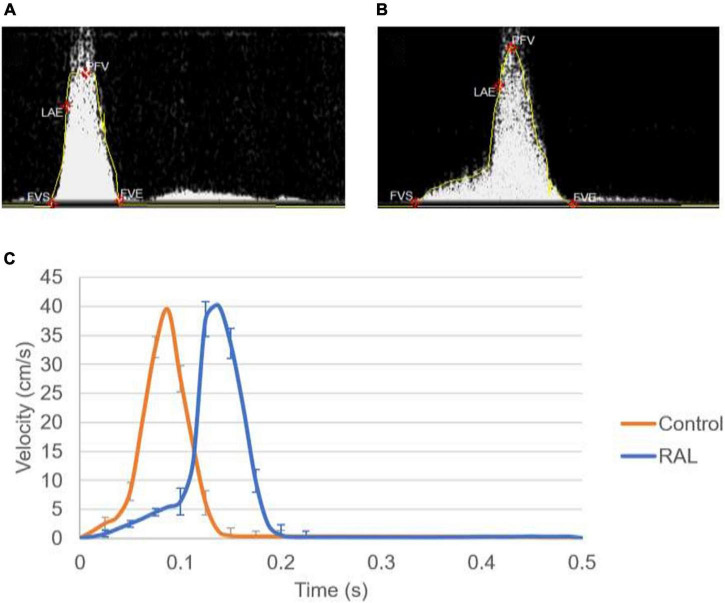
**(A)** Doppler velocity measurement for the atrioventricular (AV) canal of a sample ED5 control heart. **(B)** Doppler velocity measurement for the AV canal of a sample ED5 RAL heart. **(C)** Mean flow profiles in the AV canal of ED5 control and RAL samples.

Mean flow profiles for control and RAL hearts are presented in Figure 3. Only one peak is observed in the AV canal flow measurements of ED5 (HH27) RAL and control hearts. Up to HH23 stage, two distinct peaks can be seen in the flow profiles, but only one peak velocity can be observed for the HH27 embryos (Yalcin et al., 2011). The peak flow velocity is similar for both control and RAL hearts, indicating that RAL microsurgery does not affect the peak flow rate in the AV canal, but it does affect the instant of the peak flow with a time difference between RAL and control hearts. The instant of peak velocity is 0.05 s late for the RAL hearts when compared to the control hearts at ED5 (HH27). Our results displayed that the peak flow rates reached 40.3 cm/s at 0.1375 s for RAL hearts, and 39.5 cm/s at 0.0875 s for control hearts indicating peak flow velocity for RAL hearts are close to controls as shown in Table 1. In RAL hearts, there is a 2% increase in peak AV canal flow rate, which is considered a minor difference compared to controls with a *p*-value of 0.500.

**TABLE 1:** Time-averaged AV canal velocity, peak AV canal velocity, and ejection time for ED5 chicken embryos.

	Time averaged AV canal velocity (cm/s)	Peak AV canal velocity (cm/s)	Ejection time (s)
Controls	4.07 (0.2)	39.5 (1.99)	0.135 (0.041)
RAL	4.62 (0.25)	40.3 (2.25)	0.189 (0.065)↑
*p*-value	0.079	0.500	0.031

*The arrow pointing up indicates a significant increase and the arrow pointing down indicates a significant decrease in the values compared to the control hearts. The standard error of the mean (SEM) is given in the parentheses. The p-values are provided to indicate the statistical significance of the parameters.*

Our data can also be analyzed in terms of ejection time, a period by which the valve is open for flow (Table 1). AV canal ejection time is 0.189 ± 0.065 s for RAL and 0.135 ± 0.041 s for control hearts. The ejection time was significantly increased for RAL hearts compared to controls with a *p*-value of 0.031. The time averaged AV canal velocities during one complete cardiac cycle are 4.62 cm/s for RAL and 4.07 cm/s for control hearts with a *p*-value of 0.079, which means that RAL microsurgery has no significant effects on the mean flow rate in the AV canal of chick embryo hearts.

### The Effect of Right Atrial Ligation on Heart Morphology (Histology)

**FIGURE 4:**
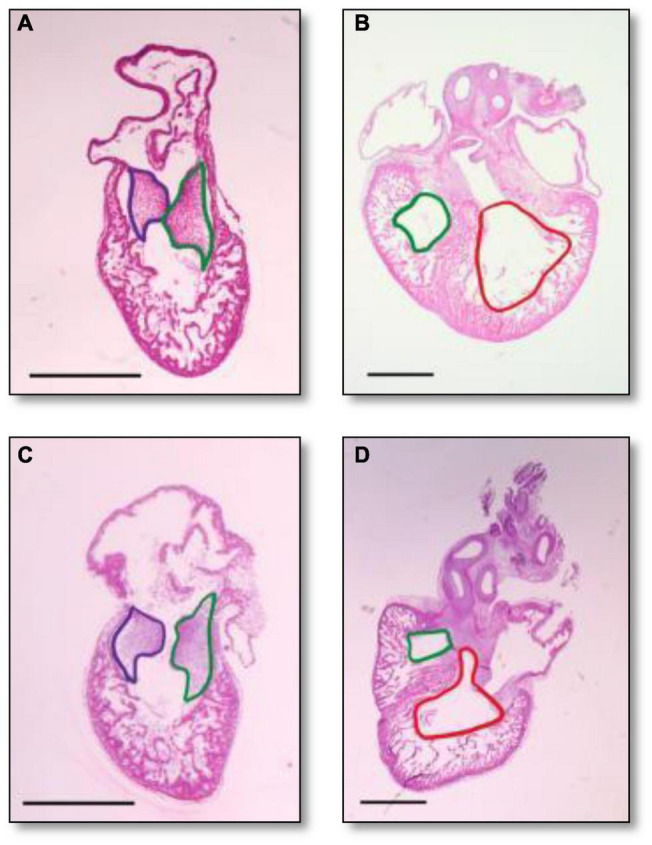
Heart section micrography of control **(A,B)** and RAL **(C,D)** samples (*N* = 3) at ED5 and ED7, respectively. At ED5, hearts of control groups displayed normal histology with normal inferior and posterior AV cushion sizes. At ED7, hearts of control groups displayed normal right and left ventricle sizes. In both control and RAL groups, purple encircles the inferior AV cushions, green encircles the posterior AV cushions, red encircles the right ventricle, and blue encircles the left ventricle. All images were stained with Hematoxylin Eosin at 4× magnification.

**FIGURE 5:**
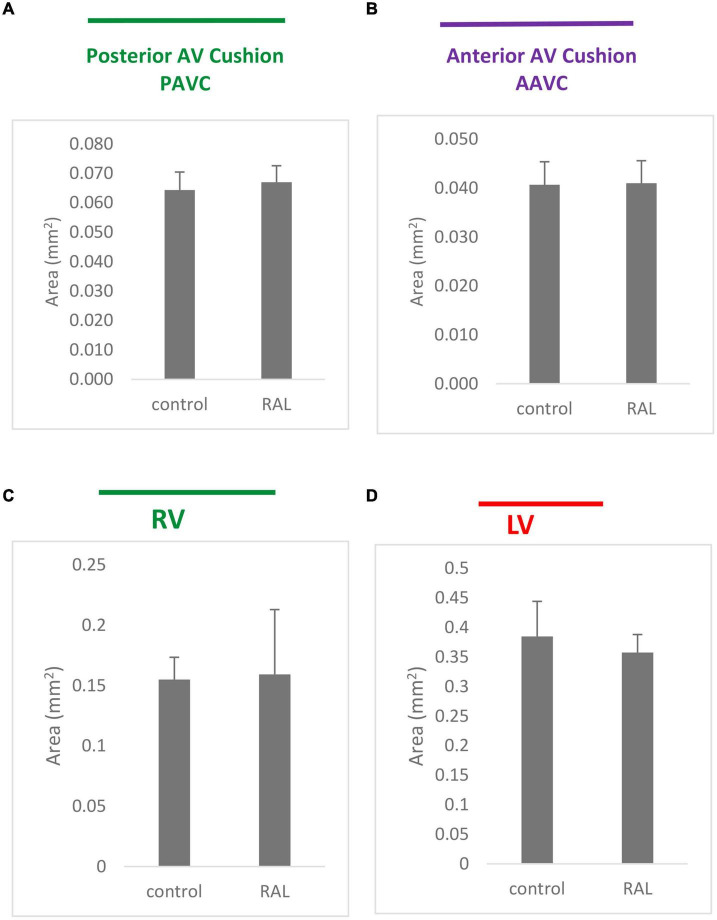
Cushion **(A,B)** and ventricle sizes **(C,D)** analysis for control and RAL hearts, **(A,B)** represent posterior AV cushions at ED5 and ED7 respectively, **(C,D)** represented inferior AV cushions at ED5 and ED7 respectively. Each bar represents mean * SD of 3 hearts.

Histological analysis makes it possible to visualize structural changes in organs. We analyzed embryos that had undergone histological analysis to assess structural differences attributable to the RAL surgical procedure. ED5 samples were analyzed (Figures 4A,C). When comparing control and RAL hearts, we observed that heart morphology and cushion sizes are very similar for both groups (encircled in green and purple). At ED7, sample hearts are septated and ventricles are almost fully developed (Figures 4B,D). Compared to controls, RAL hearts did not display any changes in ventricle sizes (encircled in red and blue). Analysis of cushion and ventricle sizes for control and RAL hearts is shown in Figure 5. At ED5, cushion sizes were similar control and ligated hearts (Figures 5A,B). Same for ventricles, comparison between control and RAL hearts did not display any significant changes in left and right ventricle sizes (Figures 5C,D).

### Micro-CT Analysis of Chamber Volumes

**FIGURE 6:**
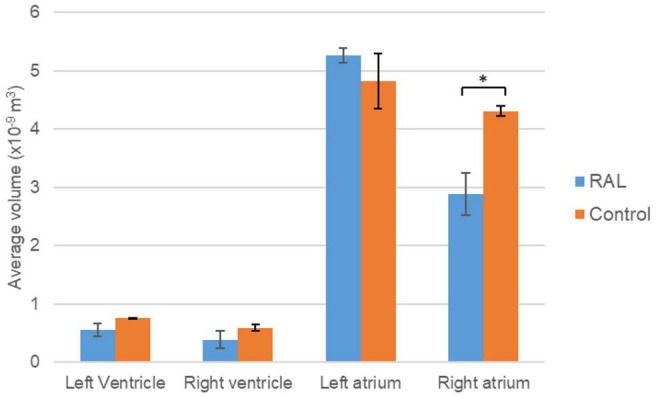
Volumetric comparison of the heart chambers for the ED5 RAL and control hearts. Asterisk (*) indicates *p* < 0.05. LV, Left Ventricle; RV, Right Ventricle; LA, Left Atria; RA, Right Atria.

**FIGURE 7:**
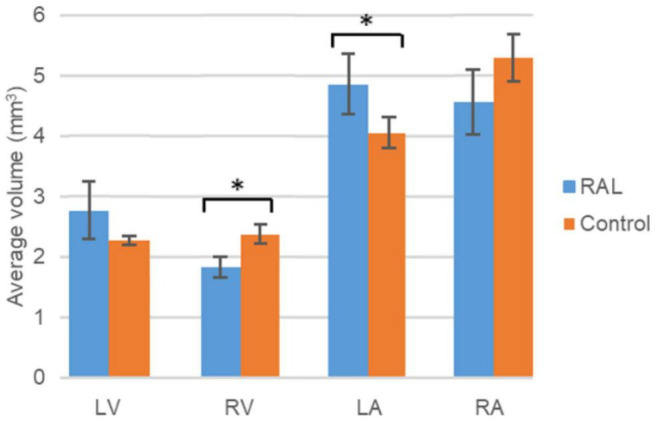
Volumetric comparison of the heart chambers for the ED7 RAL, and control hearts. Asterisk (*) indicates *p* < 0.05. LV, Left Ventricle; RV, Right Ventricle; LA, Left Atria; RA, Right Atria.

The volumes of the left and right ventricles and left and right atria are measured for RAL and control ED5 and ED7 samples. As shown in Figure 6, control ventricle sizes are smaller than atrium sizes. For ED5 control hearts, right atria, right ventricle and left ventricle volumes are determined around 4.3, 4.85, 0.6 and 0.76 mm^3^, respectively. For ED5 RAL hearts, right atria, left atria, right ventricle, and left ventricle volumes are determined around 2.9, 5.25, 0.4 and 0.56 mm^3^, respectively. At the stage of ED7, results from RAL heart chambers displayed a significant increase in the left atrium as well as a significant decrease in the right ventricle size compared to control heart chambers (Figure 7). Data from heart chamber volumes are summarized below in Table 2.

**TABLE 2:** Summary of heart chamber volumes and AV canal orifice areas with the average values and standard errors for the ED5 RAL, ED5 Control, ED7 control, and ED7 RAL hearts.

	Right atria volume (mm^3^)	Left atria volume (mm^3^)	Right ventricle volume (mm^3^)	Left ventricle volume (mm^3^)	Right AV canal orifice area (mm^2^)	Left AV canal orifice area (mm^2^)
ED5 RAL	2.887 (0.086)	5.262 (0.122)	0.391 (0.149)	0.558 (0.105)	0.17 (0.06)	0.16 (0.04)
ED5 Control	4.311 (0.366)	4.827 (0.470)	0.597 (0.059)	0.759 (0.009)	0.19 (0.02)	0.25 (0.03)
*p*-value (ED5)	0.046	0.127	0.332	0.364	0.843	0.328
ED7 RAL	4.56 (1.07)	4.87 (1.01)	1.83 (0.34)	2.76 (0.95)	0.35 (0.06)	0.36 (0.08)
ED7 Control	5.3 (0.8)	4.06 (0.52)	2.37 (0.31)	2.26 (0.15)	0.35 (0.06)	0.34 (0.08)
*p*-value (ED7)	0.139	0.026	0.017	0.558	0.254	0.123

*Standard error of the mean (SEM) is given in the parentheses. The p-values are provided to indicate the statistical significance of the parameters.*

### Results of Computational Fluid Dynamics Simulations

**FIGURE 8:**
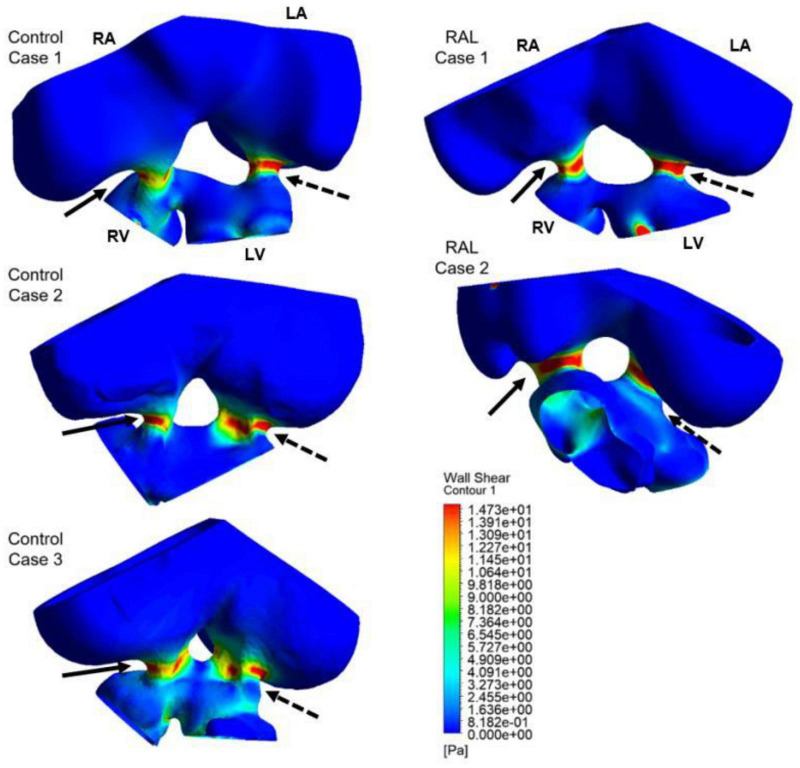
Wall shear stress (WSS) distributions at the peak AV flow rate on the ED5 RAL and control hearts. Solid and dashed arrows show right and left AV canal regions, respectively. RA, Right atria; LA, Left atria; RV, Right ventricle; LV, Left ventricle.

**FIGURE 9:**
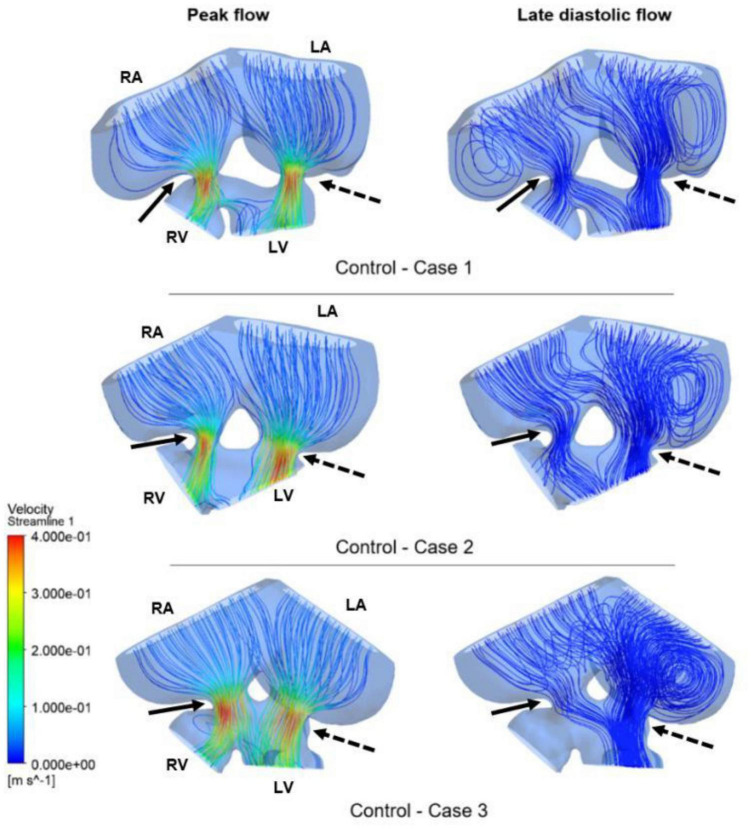
Flow streamlines in ED5 control hearts. The streamlines are shown at the peak flow rate and the late diastolic phase (at 0.4 s in the cardiac cycle) of the flow. Solid and dashed arrows show right and left AV canal regions, respectively. RA, Right atria; LA, Left atria; RV, Right ventricle; LV, Left ventricle.

**FIGURE 10:**
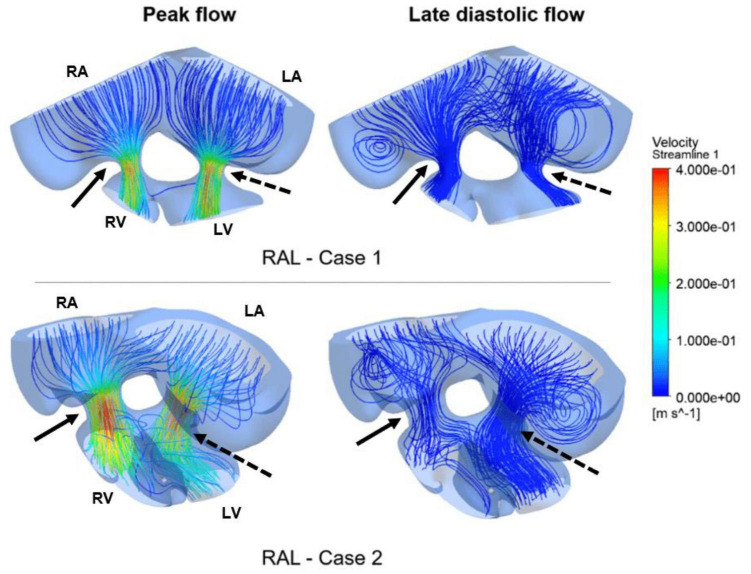
Flow streamlines in ED5 RAL hearts. The streamlines are shown at the peak AV flow rate and the late diastolic phase (at 0.4 s in the cardiac cycle) of the flow. Solid and dashed arrows show right and left AV canal regions, respectively. RA, Right atria; LA, Left atria; RV, Right ventricle; LV, Left ventricle.

Computational fluid dynamics technique is deployed to better understand blood flow velocity profiles and WSS levels in the heart. CFD simulations are performed for ED5 (HH27) control and RAL chick embryos, and particularly interested in the regions near the left and right AV canals. Figure 8 showed the exposed WSS levels at the moment of the peak AV flow rate for RAL and control hearts. Our results showed that the highest WSS levels are observed at the close proximity of the AV canals where the flow velocity significantly increases due to the reduced flow area. The WSS levels on the heart chambers are relatively lower compared to the AV canal proximity and tend to decrease with the increasing distance to the AV canals. According to this, the WSS magnitude is directly related to the frictional force exerted by the flow. Therefore, the regions with reduced flow area such as AV canals resulted in increased shear stresses on the wall. The peak of WSS reached approximately 15 Pa in the AV canals of both RAL and control hearts. In Figures 9, 10, the flow streamlines are presented for the control and RAL hearts.

Flow streamlines visualize the path that the fluid particles follow during CFD simulations. A regular and parallel distribution between the streamlines indicates a laminar flow region. The peak Reynolds number was determined around 50 in the flow domain of the modeled hearts, which is significantly smaller than the turbulence transition limit of 2000. When the hemodynamic environment in the control and RAL hearts are compared, there was no significant difference in terms of flow velocity and streamline behavior. At the peak flow moment, a regular flow distribution was observed in both control and RAL hearts with nearly parallel streamlines in the chambers. After the peak flow instant, the flow rate gradually decreased in the heart and recirculating flow vortices began to appear in the chambers depending on the reduction of flow velocity. These vortices maintained during the diastolic phase of the flow, particularly in the left and right atriums.

**FIGURE 11:**
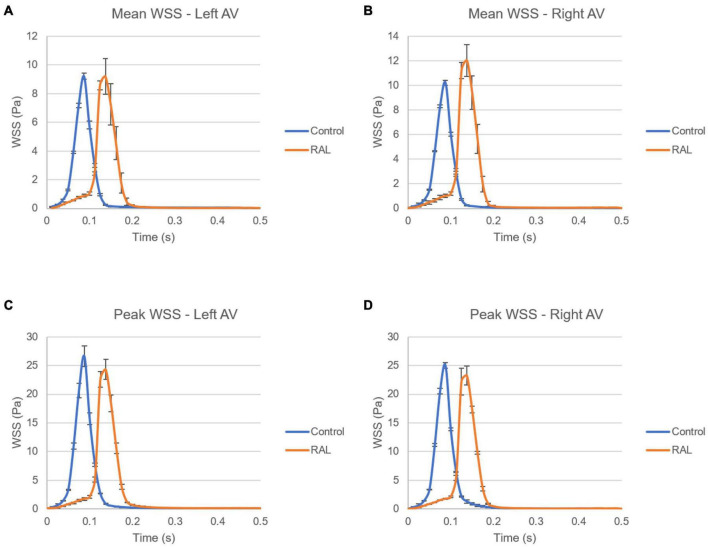
Comparison of the ED5 control and RAL hearts in terms of WSS levels in left and right AV canals. **(A)** Mean WSS in left AV canal. **(B)** Mean WSS in right AV canal. **(C)** Peak WSS in left AV canal. **(D)** Peak WSS in right AV canal.

The mean and peak WSS levels within one complete cardiac cycle were calculated and compared between the control and RAL hearts in Figure 11. The mean WSS profile in the left AV canal is nearly the same for the control and RAL hearts, with a small difference in the highest mean WSS moment. The highest mean WSS on the right AV was increased about 2 Pa for RAL hearts. This indicates that RAL did not influence the mean WSS in left AV canal, but increased the mean WSS in right AV canal. When the peak WSS levels in left and right AV canals are examined, RAL caused about 2–3 Pa reduction in the peak WSS levels on both the left and right AV canals.

**FIGURE 12:**
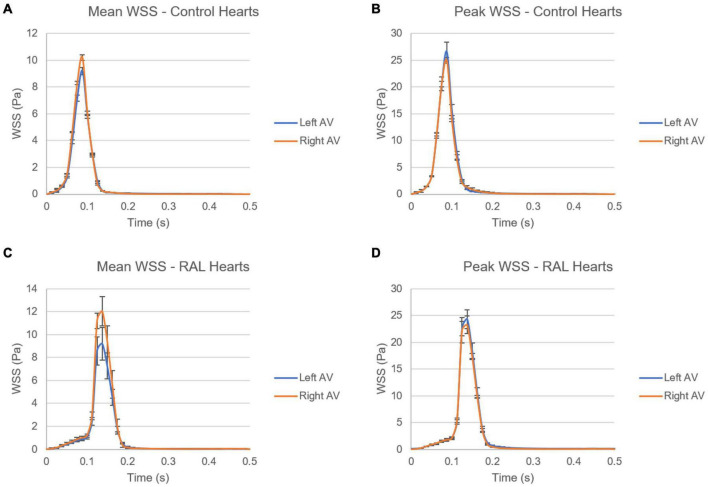
Comparison of mean and peak WSS levels on left and right AV canals of ED5 control and RAL hearts. **(A)** Mean WSS for control hearts. **(B)** Peak WSS for control hearts. **(C)** Mean WSS for RAL hearts. **(D)** Peak WSS for RAL hearts.

In Figure 12, mean and peak WSS levels are investigated by comparing the values in the left and right AV canals. In control hearts, the difference in WSS between the left and right AV canals is relatively insignificant, indicating that the WSS environment is similar on the left and right AV canals in a healthy embryonic heart. Conversely, RAL introduced an imbalance in terms of mean WSS between the left and right AV canals. There is about a ∼3 Pa difference in the maximum value of mean WSS levels on the left and right AV canals of RAL hearts. When the peak WSS levels are examined, it was observed that the peak WSS levels on the left and right AV canals are similar to RAL hearts showing that RAL did not change the balance of peak WSS between the right and left AV canals, but led to an imbalance in the mean WSS levels between the left and right sides. In our previous study (Salman et al., 2021), we observed that LAL leads to imbalance of peak WSS around 60–40% (right AV-to-left AV) at HH21 and 80–20% (right AV-to-left AV) at HH30, which indicates a greater imbalance in LAL hearts. For RAL hearts at HH27, only an imbalance around 57–43% (right AV-to-left AV) is observed for the mean WSS levels, and no significant difference is observed in peak WSS between the left and right AV canals.

The findings of heart chamber volumes and AV canal orifice areas are tabulated in Table 3 for ED5 RAL, ED5 control, ED7 RAL, and ED7 control hearts. The results of hemodynamic parameters (WSS and vorticity) determined in the CFD simulations are presented in Table 2 for ED5 control and RAL hearts.

**TABLE 3:** Summary of WSS and vorticity findings with the average values and standard errors for ED5 control and RAL hearts.

	5-day control	5-day RAL	*p*-value
Average WSS on the left AV canal (Pa)	0.825 (0.083)	0.969 (0.281)	0.649
Average WSS on the right AV canal (Pa)	0.895 (0.028)	1.211 (0.089)	0.045
Peak WSS on the left AV canal (Pa)	2.321 (0.152	2.401 (0.207)	0.779
Peak WSS on the right AV canal (Pa)	2.206 (0.091)	2.285 (0.160)	0.610
Volume averaged vorticity (s^–1^)	30.205 (4.206)	43.320 (12.345)	0.384

*Standard error of the mean (SEM) is given in the parentheses. The p-values are provided to indicate the statistical significance of the parameters.*

## Discussion

Beyond its key role in developmental stages, the heart is of major interest in many research pathways (clinical, biological, biomechanical, etc.) (Jung et al., 1973; Lucitti et al., 2005; Groenendijk et al., 2007; Salman et al., 2021). As described above, HRHS is a right-sided heart disease in which the right-sided structures are underdeveloped, restricted, or not formed. To our knowledge, no study was interested in this rare disease and its repercussion on heart morphogenesis is not well understood. Our study is the first to give an interest to HRHS using chick embryos as an animal model with a phenotype mimicking human HRHS (Sedmera et al., 2005). The lack of information on the impact of this disease on cardiogenesis compared to the huge data documented for LAL pushed us to establish a comparison between both RAL and LAL effects on chick embryo hearts. In the main part of the discussion, we will compare the severity of these 2 hypoplastic phenotypes. The aim of the present study was to assess the effects of RAL microsurgery on chick embryo hearts using selected Doppler echocardiography, histology, micro-CT, and CFD analysis techniques over periods of 5 and 7 days corresponding to the pre-septated and post-septated stages respectively.

### Right Atrial Ligation Did Not Affect AV Canal Velocities

As an integral part of heart development, blood flow is able to affect the cardiogenesis under stress condition. Doppler echocardiography allows determination of the speed and direction of blood flow using Doppler effect. In the present study, exposure of embryos to RAL did not induce any changes in the peak flow rate of the AV canal at the pre-septated stage (ED5) indicating that peak flow velocities were not a sensitive biomarker for RAL intervention. Our results are not consistent with those from LAL hearts analysis. In a previous work, our research group found that LAL microsurgery decreased the peak AV flow rate by 27.3% compared to controls (Salman et al., 2021).

Regarding the instant of the peak flow, we noted a time-difference of 0.05 s in ligated hearts compared to controls. However, the time averaged AV canal velocities were not significantly affected during one complete cardiac cycle, indicating that RAL microsurgery has no significant effects on the mean flow rate in the AV canal of chick embryo hearts. As to the LAL samples, we reported in another study a significant time-difference of 0.1 s in the peak flow instant for LAL hearts compared to controls (Salman et al., 2021) which is able to change the biomechanical environment of the heart. De Almeida et al. (2007) examined the ventricular systolic function and showed an increase in ejection fraction from 45 to 68% between ED6 and ED8 samples. In the same way, Tobita et al. (2002) demonstrated that acute intraventricular peak and end-diastolic pressure decreased in the LAL hearts with 11 and 36%, respectively compared to controls. In their analysis, a continuous decrease of the pressure remained up to stage HH27 with 18% of decrease in both peak and end-diastolic pressure compared to healthy hearts (Tobita et al., 2002). Another study by Tobita and Keller (2000) in which HH21 embryos were examined displayed that maximum AV inflow velocities decreased by 37% and remained decreased until HH25 stage by 25%. Hu et al. (2009) studied LAL hearts at 2 time points (HH21 and HH24) and demonstrated that ventricular end-systolic volume decreased by 46% and the end-diastolic volume decreased by 45% compared to controls. These authors mentioned another heart defect represented by decreased dorsal aortic stroke volumes in LAL embryos compared to controls.

### Right Atrial Ligation Did Not Induce Heart Morphological Changes

Tightening the right atrium means blocked blood flow to irrigate the right atrium chamber. To answer the question if this practice affects the normal development of the heart macrostructures, we analyzed the size of the four heart chambers as well as the size of cushions before septation (ED5). It is well important to highlight that cushions serve as primitive valves that assist in the forward propulsion of blood through the heart (Midgett and Rugonyi, 2014). They play an important role on later valve and septa formation. After the exposure of embryo hearts to RAL microsurgery, we did not mention any significant changes in the heart morphology and cushion sizes at pre-septated and post-septated stages (ED5 and ED7, respectively). We just published a new study following to the effects of LAL on heart macrostructures and defined important changes in cushion sizes that became smaller compared to healthy cushions (Salman et al., 2021). However, the differences in the effects of RAL and LAL on the cardiac morphology could be due to the difference in the LA and LV sizes. Therefore, ligating the LA leads to more reduction in blood inflow to the heart due to its large size at that stage compared to the RA. The heart seems then to be less sensitive to RAL than LAL probably due to the important physiological function of the left ventricle.

### Right Atrial Ligation Affected the Heart Chamber Volumes

The micro-CT scan images provide high resolution, efficient and accurate visualization of tissues (Doost et al., 2020). Heart sample images from ED5 and ED7 showed that RAL induced a reduction in the volume of the right atrium when compared to the healthy group. This reduction is indeed due to the ligation of the right atrium following the microsurgery. At ED7, increased volume of the left atrium was accompanied by a decrease of the right ventricle volume indicating that the obstruction of the right atrium makes different the circuit of the blood flow distribution. Nevertheless, RAL microsurgery affected the heart hemodynamics by reducing the volume of the right heart side and redirecting the blood flow to the left side. This hemodynamic redistribution is the mimetic effect of the HRHS. Likewise, we studied in a previous work about the effects of LAL on heart chamber volumes and proved important changes in LV, RV and especially RV volumes (Salman et al., 2021).

### Right Atrial Ligation and the Computational Fluid Dynamics Simulation Profile

Having the same role as blood pressure, the WSS modulate cardiac development as is required for heart formation (Midgett and Rugonyi, 2014). WSS profile is directly related to the frictional force generated by the flow. Regions with reduced flow area resulted in increased WSS due to increased flow velocities. CFD is a highly recommended computational method dedicated to compute shear stresses on the endocardium. It calculates precise blood flow velocity distributions in 3D or 4D models of the heart. Our results demonstrated similar peak of WSS in the AV canals between healthy and ligated hearts. In the left AV canal of ED5 samples, RAL did not affect both peak and mean WSS, appeared similar to healthy hearts. In the right AV canal, only the mean WSS was significantly affected by the ligation indicating that WSS unbalance occurred in the right heart side and deployed to compensate the block of the right atrium. When comparing the mean of WSS in both left and right AV canals, we noted a difference of 3 Pa, a value that is not pronounced comparing to the effect of LAL proving that RAL has no drastic effects on the WSS. The LAL microsurgery initiated an immediate imbalance of the WSS between the right and left AV canals (Salman et al., 2021). According to these authors, this WSS alteration has an important role in the development of CHDs. Analysis of the LAL hearts demonstrated an imbalance of peak WSS at ED5 and ED7 that may critically affect the development of the heart and led to structural cardiac defects (Salman et al., 2021). Chick embryos which are exposed to LAL interference displayed decreased WSS in the left side of the heart (Kowalski et al., 2014a,b).

In conclusion, RAL did not cause drastic flow disturbances compared to LAL. This microsurgery did not significantly affect cardiogenesis. Our results highlighted the ability of heart morphogenesis to take place with minimal defects compared to LAL. This may be due to lower plasticity of the right ventricle compared to the left ventricle or minimal hemodynamic alterations compared to LAL.

## Data Availability Statement

The original contributions presented in the study are included in the article/supplementary material, further inquiries can be directed to the corresponding author.

## Ethics Statement

The animal study was reviewed and approved by Warren Burggren.

## Author Contributions

MA: right atrial ligation experiments and histology. HS: CFD analysis and manuscript writing. AN: correction and synthesis of the manuscript. TK: CT scans. TS: management of CT scans and revision of the manuscript. HY: conception and monitoring of the work and revision of the manuscript. All authors contributed to the article and approved the submitted version.

## Conflict of Interest

The authors declare that the research was conducted in the absence of any commercial or financial relationships that could be construed as a potential conflict of interest.

## Publisher’s Note

All claims expressed in this article are solely those of the authors and do not necessarily represent those of their affiliated organizations, or those of the publisher, the editors and the reviewers. Any product that may be evaluated in this article, or claim that may be made by its manufacturer, is not guaranteed or endorsed by the publisher.
